# Navigating protean career paths in medical education: insights from outstanding medical educators in South Korea

**DOI:** 10.1186/s12909-024-06183-7

**Published:** 2024-10-19

**Authors:** Bora Lee, Danbi Lee, Hyekyung Shin, Sohee Park, Eunbae B. Yang

**Affiliations:** 1 Department of Dental Education, Yonsei University College of Dentistry, 50-1 Yonsei-ro, Seodaemun-gu, Seoul, 03722 Republic of Korea; 2 Department of Medicine, Yonsei University College of Medicine, 50-1 Yonsei-ro, Seodaemun-gu, Seoul, 03722 Republic of Korea; 3 Department of Preliminary Medicine, Seoul National University College of Medicine, 103 Daehak-ro, Jongno-gu, Seoul, 03080 Republic of Korea; 4 Department of Medical Education, Yonsei University College of Medicine, 50-1 Yonsei-ro, Seodaemun-gu, Seoul, 03722 Republic of Korea

**Keywords:** Medical education, Medical educator, Career development, Protean career, Qualitative study

## Abstract

**Background:**

This study explored the career development experiences of Korean medical educators who have forged their paths amid dynamic medical education landscapes. Additionally, it explored their career development process by introducing a protean career theoretical framework, that is, an individual-led career development theory.

**Methods:**

This study employed Interpretative Phenomenological Analysis (IPA) to gather in-depth insights regarding the experiences of medical educators who have successfully built their careers in Korea. Semi-structured interviews were conducted with nine medical educators to investigate the significance of these experiences. The emerging themes were categorised based on the protean career theory during data analysis.

**Results:**

The findings revealed that medical educators navigated their careers in line with the protean career development model, characterised by protean career orientation, process and outcomes. Their experiences in the medical education domain were aligned with eight factors of the protean career model – self-direction, intrinsic work values, awareness, adaptability, agency, subjective career success, objective career success and organisational commitment.

**Conclusion:**

In the context of less structured career pathways and a rapidly evolving regional environment, medical educators have developed individual-driven careers with self-direction and intrinsic values, formed their identities and demonstrated flexibility and proactive strategies. Hence, the protean career model successfully explains the characteristics of self-directed career development for medical educators, while emphasising the need for organisational support.

**Supplementary Information:**

The online version contains supplementary material available at 10.1186/s12909-024-06183-7.

## Background

Perceived as partially ambiguous, the identity of a ‘medical educator’ is often defined based on activities and roles [1]. A medical educator is a person who works in research, management and administration and is engaged in scholarship in the medical education field. Unlike a ‘clinical teacher’, a medical educator is not necessarily involved in clinical teaching, although the two identities may overlap [2]. A medical educator’s role encompasses content expertise, enhancing the education provided by others, and being an academic expert in creating and disseminating research-based knowledge [3]. Harden defined 12 roles of a medical teacher and a later study regarding the self-definition of a medical educator identified five additional roles [4, 5]. These varied definitions and roles are due to the wide-ranging educational needs of the field implying that medical educators’ career development progresses dynamically with diverse experiences in medical education and healthcare.

Throughout their careers, medical educators experience conflict between their identities as clinicians and educators and education-related values influence their identity as medical educators [6]. They struggle to maintain their identity as educators and develop stable careers amid competing career demands [1, 3]. A clinician or researcher’s role is often prioritised over a medical educator because the former receives better support and greater social capital [6–8]. The changes required to acquire what is perceived as an inferior and ambiguous identity lead to stress and inhibit further participation by junior educators, regardless of programme and organisational support [1, 9]. In these circumstances, understanding the potential of medical education, networking between medical educators, mentoring and teaching enjoyment are important for the professional development of medical educators [1]. 

Within the limited research regarding the identity, roles and career development of medical educators, this study addresses the research question– How do medical educators shape their professional identities and roles throughout their careers and what are the challenges and opportunities they encounter? Hence, this study aims to deepen the understanding of Korean medical educators’ identities and career development, often recognised as diverse and ambiguous. Furthermore, it highlights the unique challenges and opportunities they face in this process.

### Theoretical background

**Fig. 1:**
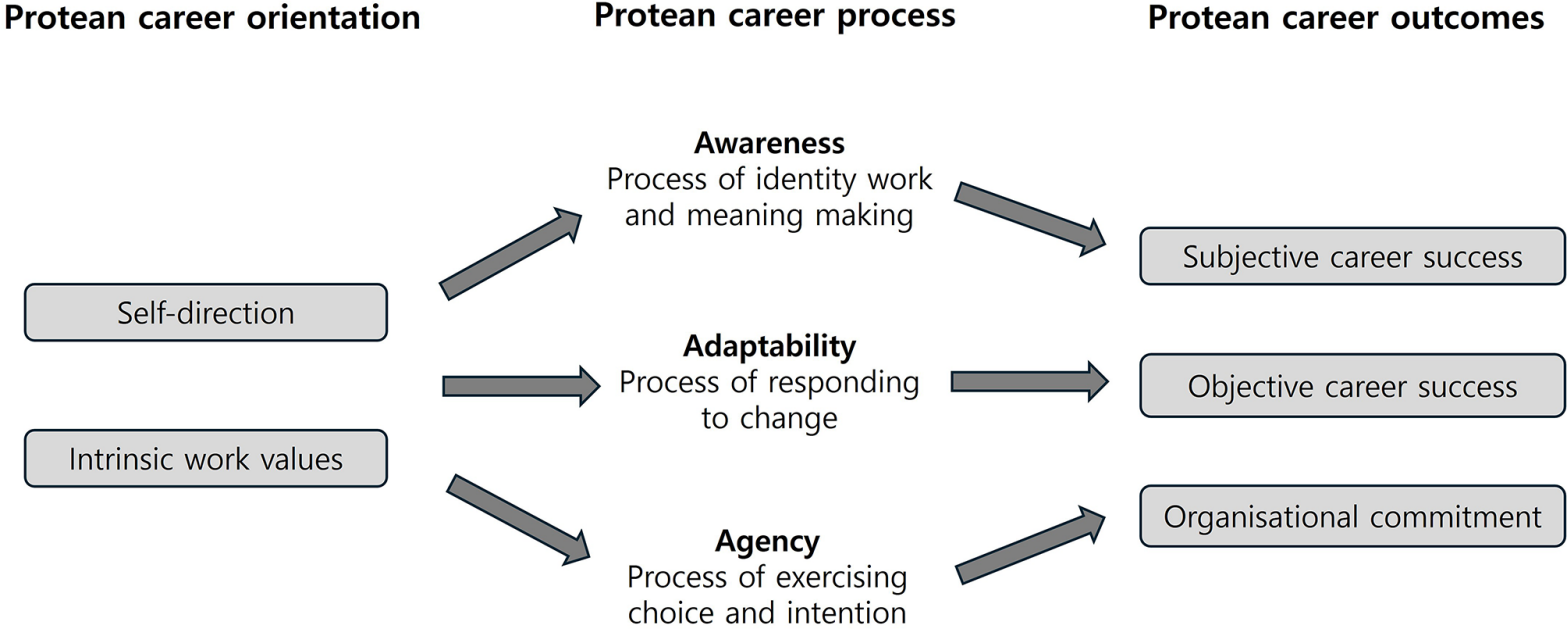
Key mechanisms in the protean career process [14]

The traditional view of career development believes that an individual’s career grows and develops within an organisation and individual career development is often explained within the framework of the organisational structure [10]. However, a protean career, which takes its name from Proteus, a shape-shifting Greek God, is an individual-oriented career development idea, which encompasses changes in work, occupation and organisation to suit the situation [11]. A protean career is individual-led, based on the goals they set throughout their life, and values psychological success over objective success (e.g., wages, promotions and social power) [12]. Therefore, success can be achieved in diverse ways in a protean career because it is based on individual choices and values. Furthermore, a protean career is self-directed, rather than organisation-directed, and is motivated through continuous learning and identity changes [12]. It comprises individual experiences through learning, training and working in various organisations [13]. The protean career mechanism consists of three key components – orientation, process and outcomes (Fig. 1) [14, 15]. Orientation is defined by self-direction and intrinsic values, while the process involves awareness, adaptability and agency. Outcomes include both subjective and objective career success and organisational commitment. Each of these elements is explored in greater detail in the results section for better readability and understanding.

### Context

There are 40 accredited medical schools in South Korea, all located within universities. As of 2019, 11,111 full-time tenured faculty members were working at the medical schools, of whom more than 500 medical educators were members of the Korean Association of Medical Colleges (KAMC), Korean Society of Medical Education (KSME) and Korean Institute of Medical Education and Evaluation (KIMEE), conducting administrative work and educational research to enhance medical education quality [16]. Korea’s medical educators have experienced numerous changes in a short period and have successfully solved various issues regarding the country’s medical education by introducing foreign medical education systems and curricula [17]. Based on their efforts, the number of schools introducing integrated curriculum and problem-based learning increased and competency-based (outcome-based) education curricula were developed and implemented [17, 18]. Additionally, the evaluation and accreditation system for Korean medical schools was implemented in 1996 [19]. The introduction of practical exams provided an opportunity to improve medical education quality [20]. 

## Methods

This study employed Interpretative Phenomenological Analysis (IPA) to gain an in-depth understanding of medical educators’ experiences who have successfully sustained their careers in Korea and uncover the meaning of their experiences through semi-structured interviews. During data analysis, the emerging themes were categorised based on the protean career theory.

### The qualitative approach: IPA

Phenomenology focuses on understanding the meaning of participants’ experiences through their unique life experiences [21]. IPA adheres to the interpretative tradition and is grounded in three primary theoretical foundations [22]. First, phenomenology, originally articulated by Husserl, is a philosophical approach that describes experiences from the perspective of the experience itself, rather than through pre-existing theoretical biases [23]. Second, IPA recognises the interpretative nature of this approach, that is, humans are naturally inclined to make sense of their experiences, therefore, in IPA, the researcher interprets how the participants make sense of what is happening to them. Third, IPA, characterised by its idiographic focus, examines each individual’s experience in detail before making broader generalisations. Moreover, it encourages researchers to utilise their theoretical knowledge to perform inductive data analysis, emphasising individual cases and enabling a systematic and in-depth interpretation of participants’ experiences [24]. This study, incorporating a protean career theoretical framework, employed IPA to explore the career development experiences of medical educators, understand their perspectives and derive meanings from their experiences.

### Data collection

This study was approved by the Institutional Review Board (IRB) of Yonsei University Health System, Severance Hospital under protocol number 4-2023-0231. Informed written consent was obtained from all participating medical educators after explaining the study’s purpose and background and its confidentiality and compliance with ethical standards.

Purposive sampling was employed to select the study’s participants. The selection criterion included extensive experience in the medical education field in South Korea, recipient of awards within the last five years (between 2017 and 2022) from major medical education-related organisations in South Korea, specifically the KAMC and the KSME, and accomplished career success and holding leadership positions at medical education institutes.

The specific awards considered for participant selection were the Myung-gok Medical Education Award (awarded by the KSME for significant contributions to the development of Korean medical education) [25], the Han-gok Medical Education Academic Award (awarded by the KSME in both research and education categories for outstanding academic achievements related to medical education) [25] and the Medical Education Innovation Award (awarded by the KAMC to professors at medical schools or teaching hospitals who have led creative innovation and contributed significantly to the development of medical education) [26]. 

As of 2022, 19 individuals were awarded the Han-gok Medical Education Academic Award, five were honoured with the Myung-gok Medical Education Award and five were conferred the Medical Education Innovation Award. Among them, three were multiple awardees. Additionally, from 2017 to 2022, nine awardees were identified as possessing leadership experience at medical schools or institutions related to medical education. Thus, these nine candidates were invited to participate in the study. All of them agreed.

In-person or online semi-structured interviews were conducted based on the participant’s convenience. Regardless of the interview mode, all the interviews adhered to similar time frames, procedures and criteria to maintain consistency. This approach allowed data collection uniformity, gave due regard to the participants’ preferences and made the interview process convenient. Of the nine participants, four were interviewed in person and five online. The questions, designed to elicit comprehensive responses, are detailed in Appendix 1. Each interview lasted approximately 1.5–2 h, was audio-recorded and transcribed verbatim for detailed analysis.

### Data analysis

The qualitative analysis entailed repeatedly reading the data, rephrasing, developing emergent themes and systematically analysing the interconnected themes by applying relevant career development theories [24]. The researchers applied the protean career theory, recognising its emphasis on the self-directed and value-driven aspects of career development. By providing a suitable framework for understanding the dynamic and individualised career paths of medical educators, the protean career theory assisted in organising and interpreting the themes that emerged throughout the study. Finally, the researchers evaluated the validity of integrating each case under similar superordinate themes and identified common patterns among participants’ experiences [24]. 

To establish reliability and validity in the textual analysis of qualitative data, triangulation is suggested for correlating people, time and space (data triangulation), correlating findings from multiple researchers (investigator triangulation), correlating multiple theories (theory triangulation) and using multiple data collection methods (methodological triangulation) [27]. This study, for data triangulation, attempted to conduct a context-rich interpretation of life experiences by understanding the period in which the participants worked as medical educators. For investigator triangulation, three researchers, including a professor with extensive knowledge of South Korean medical education, participated in the interviews and provided feedback during the interpretation process. For methodological triangulation, online searches were performed and data collected on the participants’ careers as doctors or medical educators, newspaper articles about their medical education activities and their published medical education-related papers.

The interviews were conducted in Korean and the translation was carried out in the final data analysis stage. Given the strong mutual influences between language and thinking and the recommendation to ‘stay in the original language as long and as much as possible’, ongoing reflection and back translation were implemented during the translation phase to refine the translated material and accomplish a nuanced meaning [28–30]. To ensure accuracy, the translation was reviewed by the three researchers (B Lee, D Lee, and EB Yang), followed by a final review by a native English speaker to enhance linguistic precision.

## Results

Individual interviews were conducted with nine participants in May – June 2023. The participants’ characteristics are presented in Table 1. Using protean career theory as a framework, the following themes emerged.

**Table 1:** Characteristics of participants

Medical doctor qualification	Awards of medical education			Area of specialties			Experiences in medical education-related institutions	
	Myung-gok Award*	Han-gok Award*	Innovation Award#	Basic science	Clinical science	Education	Dean of medical school	Head of national or international institution¶
O	O	-	-	O	-	-	O	-
O	O	-	O	-	O	-	O	-
O	O	-	O	-	O	-	-	O
O	O	-	O	-	O	O	O	O
O	O	-	-	-	O	-	-	O
O	-	O	-	-	O	-	O	-
O	-	O	-	-	O	-	O	-
O	-	-	O	O	-	O	-	O
O	-	-	O	-	O	-	O	-

^*^ Awarded by the Korean Society of Medical Education

^#^ Awarded by the Korea Association of Medical Colleges

^¶^ International Academic Association, the Korean Institute of Medical Education and Evaluation (KIMEE), Medical Education Training Institute, amongst others

### Protean career orientation

Protean career orientation is a career approach characterised by self-direction where individuals desire to be agentic, in control of their careers and value-oriented. Intrinsic values guide their career decisions [14]. Here, the person rather than an organisation or others, is in charge, leading to self-direction, that is, an individual’s independence from external control or influence [14]. An individual’s interaction with intrinsic values leads to career selection and transition, whereby, they actively create their meaning rather than adhering to externally defined meanings [14]. 

### Self-direction

Participants’ self-direction in their career development was evident through their self-directed learning. Understanding the importance of continuous self-development for enhancing their expertise as medical educators, they have consistently engaged in self-directed learning:

*‘I think the most important thing is the ability to identify essential educational issues and to learn the related educational principles on your own. I have always done that by studying through self-directed learning and teaching’. (Participant B)*

The participants pursued self-directed learning by pioneering related study routes. Although they lacked external faculty development support, they pursued self-directed learning and growth as medical educators, gradually building academic expertise in the field. Specifically, they created self-learning opportunities by attending overseas conferences with limited support:

*‘I wasn’t expecting that kind of support…Studying was something I could do by collecting resources on my own. I organised my schedule and went to educational conferences’. (Participant C)*

### Intrinsic work values

Participants recognised that the value of medical education lies in its ability to nurture physicians who provide compassionate patient care and contribute to society. They believed that medical education could ‘establish good doctors with real-world problem-solving ability, that is, humanistic and socially responsive, who are interested in “solving social issues”’ (Participant A). An understanding of these intrinsic values is crucial for sustaining a career as a medical educator because a superficial approach of viewing work values as ‘giving better lectures’, ‘becoming more popular with students’, or ‘securing a job’ could lead to burnout (Participant A). Nonetheless, they believed that medical educators who possess a professional calling and an educational philosophy can ‘continuously dedicate themselves’ and bring ‘change in the field of medical education’ (Participant D). The participants agreed that, for medical educators, having an ‘educator mindset’ is more important than possessing ‘educational skills’ and the associated ‘educational philosophy’ (Participant F).

Some of the experiences identified by the participants played a significant role in shaping their values regarding medical education and their role as medical educators. Based on their experiences as students, residents and professors, the participants developed a critical perspective on medical education and the importance of teaching and learning methods, which highlighted the importance they placed on a medical education career. For example, one participant detailed the problems they faced as a student with ‘memorisation-based education’. However, after encountering new teaching methods, such as ‘project-based learning’, during an overseas training opportunity, they strengthened their resolve to improve medical education and decided that ‘I will dedicate myself to medical education instead of research’ (Participant A). Another participant was motivated towards medical education after encountering an ‘excellent teacher’ during their residency training who enhanced their interest in medical education (Participant C). Recognising the problems with memorisation-based education while teaching students, a participant observed their ‘inability to connect what they have studied with clinical problems’. This experience motivated them to explore and apply new teaching methods, eventually realising that Problem-Based Learning (PBL) should be their target approach, despite being unaware of its theory initially (Participant E).

### Protean career process

The protean career process refers to an individual’s capability to demonstrate a protean career orientation constituting identity awareness, adaptability and career agency [12, 14]. Identity awareness implies having a clear sense of one’s personal identity and value and high self-awareness. These are the key meta-competencies associated with protean career orientation [31]. Adaptability is the characteristic of adjusting to a changing environment [32, 33]. Agency refers to the intentional act of making things happen through an individual’s actions [34]. 

### Awareness

By recognising the importance of identity in career development, the participants could define their identity and role as medical educators. Consequently, their performance within the organisation improved, thereby, continuing their career development:

*‘Who is a medical educator?…If this isn’t clear*,* people may get confused. So*,* while carrying out activities*,* the territory of medical education must be clearly defined’. (Participant G)*

A participant encountered role conflict due to ‘dual positioning of the job’, however, observing senior medical educators and their dedication to work served as motivation for maintaining a medical education career (Participant D). Additionally, recognising the commonalities between clinical practice and medical education led to the discovery of similarities in the roles of a clinical physician and a medical educator, which is ‘100% the same’, facilitating the integration and development of both roles (Participant F). The identities of clinicians and medical educators were not mutually exclusive, instead developed through convergence. Furthermore, their activities showed a synergistic effect that was mutually beneficial:

*‘Did the education have any influence on my clinical activities? Of course*,* it certainly did. I think both sides can provide synergy with each other. I think it would be better to just have fun and work hard at it. Both influence each other positively*,* I think’. (Participant G)*

### Adaptability

The participants highlighted the need for the evolution of medical education based on social needs and the medical environment. To achieve this, it is necessary to examine medical education at a ‘global level’ rather than having a ‘tunnel view’ (Participant B). Additionally, medical educators are expected to ‘always look ahead’ and ‘try to figure out future changes at least 10 years in advance’ (Participant A). A flexible and macro-level mindset is essential for medical educators due to the dynamic healthcare environment:

*‘I believe that medical education has been changing depending on the medical environment and is not fixed but flexible. That is why medical educators must work with insights and a broad perspective that allows them to see the whole picture’. (Participant E)*

In the Korean context, the participants encountered demands for educational changes, such as improvements in the national exam, the introduction of practical exams like OSCE (Observed Structured Clinical Examination) or CPx (Clinical Performance Exam) and the adoption of computer-based methods. When these educational changes first emerged, they ‘didn’t know what they were’ (Participant D). However, to adapt to these changes, they engaged in self-directed learning to study the ‘educational philosophies and terms’ related to new assessment methods, such as OSCE and CPx, and ‘tried to learn educational theories’ (Participant D).

### Agency

Additionally, the participants acknowledged that having strategies for inducing and implementing changes proved crucial in shaping and managing career development as medical educators. They perceived that educational innovation was possible by changing the professors’ ‘mindset’:

*‘I think that changing the mindset of professors is the most important thing. If the professor’s mindset changes*,* we will be able to build a proper system. However*,* if the mindset does not change*,* we will end up failing’. (Participant B)*

They encountered resistance due to varied wills and values among faculty members while attempting to implement changes in medical education. Faculty members often resisted change because they ‘had not experienced it before’ (Participant I). In such cases, it is important to ‘explain why it is needed’, ‘communicate the reasons continuously’ and ‘prepare them well to experience success’ (Participant I). Additionally, regularly holding one-on-one or group discussions to engage faculty helped ‘facilitate their understanding of current medical education issues from educational perspectives’ and ‘foster their interest in education’ (Participant A).

Additionally, medical educators recognised the importance of communication with students for applying new curricula or teaching and learning methods. They shared the school’s policies and explained the educational agenda to students while listening to their needs:

*‘We had a meeting with the student representatives once a month and talked about the school policies actively. Students discussed their needs every time and presented them to us clearly. We always held an information session for the whole student body at the beginning of every semester’. (Participant D)*

Changes enacted through formal institutions by implementing accreditation processes proved effective in improving education quality to ‘share what was the national or international standard and the future direction of medical education’ (Participant F). Additionally, ‘when such changes are unlikely to occur voluntarily’, legislated accreditation standards facilitated the necessary transformations (Participant C). Thus, medical educators’ career activities, through formal institutions, proved effective in inducing changes within individual and across medical schools nationwide. Although medical educators faced significant resistance when working with formal organisations, they overcame these challenges by collaborating with the ‘core members’ and ‘working jointly to navigate the difficulties’ (Participant F).

### Protean career outcomes

Protean career outcome includes subjective and objective career success and organisational commitment. Subjective career success refers to the psychological experience of success based on an individual’s goals or expectations, while objective career success refers to observable and measurable achievements [35–37]. 

### Subjective career success

The participants found their role as teachers in medical education significant. As both medical educators and teachers, their greatest joy lay in meeting students and nurturing them into good physicians:

*‘I think it’s the most important thing to have fun in medical education. I’m very lucky to have been involved in medical education. There is no better job than that of a medical educator in the pursuit of fostering good physicians’. (Participant F)*

The participants experienced psychological satisfaction and a sense of reward by fostering student growth and learning. For example, Participant F felt satisfied when teaching students how to ‘explain medication to elderly patients’, which improved their ‘communication skills’ and positively changed ‘students’ attitudes and behaviours’. Additionally, Participant D felt immense gratification upon seeing ‘enthusiastic reactions’ from students after introducing PBL, observing that ‘students enjoyed solving problems on their own rather than simply being given knowledge’. These examples illustrate that the medical educators’ subjective career success is derived from both teaching knowledge or skills and witnessing essential changes in students, such as their attitudes and behaviours towards patients, and their enthusiasm for learning.

### Objective career success

All the participants were conferred social recognition through awards for their contributions to the field of medical education and held leadership roles in relevant organisations. They successfully organised large-scale international medical education conferences for academic collaboration, with ‘participation from more than 800 medical educators from 57 countries globally’ (Participant C). Additionally, they drew on their experience in medical education within South Korea to advance medical education in other countries, advising ‘basic medical science education development’ (Participant B) or ‘clinical education improvement, portfolio development, student learning assessment, and curriculum design and evaluation’. (Participant A).

Forming networks was considered an objective career success. While developing their careers as medical educators, they formed social and academic networks to share experiences and knowledge to solve problems and induce change in medical education:

*‘I thought it is necessary to create a group of people who love medical education*,* who are passionate and committed to medical education*,* to support each other by sharing theoretical background knowledge for practical application. By learning and working together in medical education*,* we learned about each other’s challenges and became very close’. (Participant H)*

### Organisational commitment

Participants emphasised the importance of organisational trust for medical educators to experience early career success, ‘the trust and encouragement from the faculty community significantly influence their career decisions as they plan their future’ (Participant I). Moreover, to sustain their careers without burnout, they recognised the significance of experiencing ‘small successes’ and emphasised the need to ‘provide opportunities for developing careers from small successes and engaging in higher-level decision-making’. (Participant D).

The career development of medical educators requires a supportive environment for medical education, along with various forms of support for faculty development, to provide learning opportunities:

*‘It is necessary to create an atmosphere that supports medical education. This support may include sending for training programmes*,* creating a fund*,* or supporting educational research. Additionally*,* medical educators need to be given opportunities to frequently participate in medical education-related meetings and conferences’. (Participant G)*

## Discussion

This study invited medical educators who have pioneered changes and innovations in South Korea’s medical education to explore their career development experiences. Furthermore, by employing the protean career conceptual framework, it attempted to explain the characteristics of medical educators’ career development.

For medical educators, personal goal-setting and self-direction are important because, unlike other roles, their role lacks clarity [7]. This study revealed medical educators to be self-directed learners who participated in relevant overseas conferences and training despite insufficient support. Furthermore, their intrinsic value was based on their philosophy and sense of calling towards education and the significance of education and medicine in creating competent doctors. These findings are consistent with previous research where the main motivation for clinicians to participate in medical education stemmed from their interest in medical education through their experience as learners, along with the intrinsic motivation to provide students with a better learning experience [7]. From the perspective of protean career development, it is noteworthy that intrinsic value, when combined with self-direction, makes people more responsive to change; however, without self-direction, it can lead to rigid career orientation or hinder career proactivity [11]. Additionally, the combination of self-direction and intrinsic value in medical educators served as a driving force in shaping and guiding their careers.

The protean career process, comprising identity awareness, adaptability and career agency, was identified in the career development process of medical educators. The participants acknowledged their dual identities as experts in their original specialities and as medical educators. By observing their role models, the participants found synergies between clinical practice and education. These findings are consistent with previous research, which shows that medical educators, with a background in clinical practice, duly perform their roles, while preserving their identity as clinicians [3, 7]. This demonstrates the nonconflicting nature of these roles, instead, their clinical experience served as a valuable cultural capital in their career development.

Adaptability is a fundamental value that medical educators should endorse and practice [32, 33]. It encompasses modifying educational programmes, teaching styles, priorities and content based on changes in learners or educational environments [38]. Participants emphasised the evolution of medical education in response to changing social needs and the medical environment by adopting a global perspective and a forward-thinking approach. It is significant to note that flexibility and broad insight are vital for adapting to changes, such as new examination methods and educational philosophies.

Agency is linked to proactive behaviour, high work engagement, positive career experiences, career growth, job satisfaction, organisational commitment and subjective career success [39–41]. In this study, the career agency was involved in implementing changes in medical education by altering the faculty’s mindset, improving communication with students and pursuing macro-level changes via official institutions. Understanding change management principles and adopting appropriate strategies is significant for managing change in medical education [42]. For example, implementing curriculum reform necessitates sharing a common vision and transparently involving faculty and students to create ownership and facilitate change implementation [43]. 

Based on previous literature, subjective career success includes pride in achievement, intrinsic job satisfaction, self-worth, commitment to work role or institution, fulfilling relationships and moral satisfaction. For objective career success, previous literature suggests status and rank (hierarchical position), material success (wealth, property and earning capacity), social reputation and regard and prestige [44]. Although there were limitations in comprehensively identifying all the factors related to subjective and objective career outcomes, subjective satisfaction from realising intrinsic values and objective involvement in international networks were important career outcomes. In previous studies, medical educators have valued rewards, such as the enhancement of students’ insight and professional progress and the sense of satisfaction derived from their roles [45–47]. Additionally, with globalisation and internationalisation becoming central agendas in medical education, the role and influence of medical educators are bound to expand, suggesting that international activities will play an increasingly pivotal role for future medical educators [48]. 

Organisational commitment is influenced by an individual’s mindset, which includes normative (perceived obligation to stay), continuance (perceived cost of leaving) and affective (emotional attachment to the organisation) commitment [49, 50], the affective component being its essence. Numerous longitudinal studies report a significantly high negative correlation between affective commitment and turnover, reducing workplace stress levels, burnout and emotional exhaustion [49, 51, 52]. Affective commitment, which correlates positively with work continuity and job satisfaction, is positively related to perceived organisational support and trust [53, 54]. This study’s participants highlighted that organisational trust and encouragement are crucial for early career success in medical education and emphasised the significance of opportunities for gradual involvement in higher-level decision-making following small successes for sustaining careers. Additionally, organisational support, encompassing faculty development, educational facilities, formal career paths, university support, leadership and teaching environment, is vital for the career development of medical educators [55]. The participants underscored the importance of providing both organisational trust and practical support for advancing medical educators’ careers.

In South Korea, medical educators, demonstrating self-direction and intrinsic values, have effectively driven and implemented significant changes in medical education, showcasing their ability to sustain and advance their careers. The protean career model, which emphasises flexibility and adaptability, aligns with the evolving field of medical education and supports lifelong individual-driven learning. However, this model is subject to certain limitations, such as the lack of a structured career path and potential career uncertainty, which can pose challenges when integrating it with traditional career structures in medical schools. In the South Korean context, the need to balance flexibility with institutional support is vital for the career development of medical educators.

Future research should explore the extent to which the protean career development model effectively explains the career progression of medical educators, while considering cases that do not align with the model and addressing critical perspectives to provide practical implications for structuring medical education and supporting early-career medical educators.

## Conclusion

In the context of less structured career pathways and a rapidly evolving environment specific to Korean medical education, medical educators have developed individually driven careers characterised by self-direction and intrinsic values, shaping their identities and demonstrating flexibility and proactive strategies. The protean career model elucidates the self-directed nature of career development, observed among these educators, and underscores the importance of organisational support.

## Electronic supplementary material

Below is the link to the electronic supplementary material.

Supplementary Material 1

## Data Availability

The datasets used and/or analysed during the current study are available from the corresponding author on reasonable request.

## References

[1] Sabel E, Archer J. Medical education is the ugly duckling of the medical world and other challenges to medical educators’ identity construction: a qualitative study. Acad Med. 2014;89(11):1474–80. 10.1097/acm.0000000000000420. 25054418 10.1097/ACM.0000000000000420

[2] Bleakley A, Bligh J, Browne J. Medical Education for the future. Dordrecht Heidelberg London New York: Springer; 2011.

[3] Hu WCY, Thistlethwaite JE, Weller J, Gallego G, Monteith J, McColl GJ. It was serendipity’: a qualitative study of academic careers in medical education. Med Educ. 2015;49(11):1124–36. 10.1111/medu.12822. 26494065 10.1111/medu.12822

[4] Harden RM, Crosby JAMEE, Guide No. The good teacher is more than a lecturer - the twelve roles of the teacher. Med Teach. 2000;20(4):334–47. 10.1080/014215900409429.

[5] Nikendei C, Ben-David MF, Mennin S, Huwendiek S. Medical educators: how they define themselves - results of an international web survey. Med Teach. 2016;38(7):715–23. 10.3109/0142159x.2015.1073236. 26383184 10.3109/0142159X.2015.1073236

[6] Kumar K, Roberts C, Thistlethwaite J. Entering and navigating academic medicine: academic clinician-educators’ experiences. Med Educ. 2011;45(5):497–503. 10.1111/j.1365-2923.2010.03887.x. 21486325 10.1111/j.1365-2923.2010.03887.x

[7] Bartle E, Thistlethwaite J. Becoming a medical educator: motivation, socialisation and navigation. BMC Med Educ. 2014;14(1):110. 10.1186/1472-6920-14-110. 24885740 10.1186/1472-6920-14-110 PMC4047547

[8] Dahlstrom J, Dorai-Raj A, McGill D, Owen C, Tymms K, Watson DA. What motivates senior clinicians to teach medical students? BMC Med Educ. 2005;5:27. 10.1186/1472-6920-5-27. Epub 20050718. 16022738 10.1186/1472-6920-5-27 PMC1185542

[9] Hu WC, McColl GJ, Thistlethwaite JE, Schuwirth LW, Wilkinson T. Where is the next generation of medical educators? Med J Aust. 2013;198(1):8–9. 10.5694/mja12.11654. 23330743 10.5694/mja12.11654

[10] Schein VE. Individual Power and Political Behaviors in Organizations: an inadequately explored reality. Acad Manage Rev. 1977;2(1):64–72. 10.5465/amr.1977.4409169.

[11] Briscoe JP, Hall DT. The interplay of boundaryless and protean careers: combinations and implications. J Vocat Behav. 2006;69(1):4–18. 10.1016/j.jvb.2005.09.002.

[12] Hall DT. Careers in and out of Organizations. Thousand Oaks (CA): Sage Publications Ltd.; 2002.

[13] Hall DT, Mirvis PH. Careers as lifelong learning, the changing nature of work. Hoboken (NJ): Jossey-Bass/Wiley; 1995.

[14] Hall DT, Yip J, Doiron K. Protean careers at work: self-direction and values orientation in psychological success. Annual Rev Organizational Psychol Organizational Behav. 2018;5:129–56. 10.1146/annurev-orgpsych-032117-104631.

[15] Hall DT. Careers in Oraganizations, Glenview (IL). Scott Foresman & Co.; 1976.

[16] Yang EB, Lee TS, Cho MJ. Current status and performance evaluation systems of Faculty in Korean Medical Schools. Korean Med Educ Rev. 2019;21(1):41–50. 10.17496/kmer.2019.21.1.41.

[17] Kim KJ, Kee C. Reform of medical education in Korea. Med Teach. 2010;32(2):113–7. 10.3109/01421590903197043. 20163225 10.3109/01421590903197043

[18] Roh H, Rhee BD, Lee JT, Bae SK. Development of Task-based learning outcomes according to clinical presentations for clinical clerkships. Korean J Med Educ. 2012;24(1):31–7. 10.3946/kjme.2012.24.1.31. 25812789 10.3946/kjme.2012.24.1.31 PMC8814538

[19] Lee ST, Yang EB. Analysis of the degree of social accountability in accreditation standards for basic medical education. Korean Med Educ Rev. 2023;25(3):273–84. 10.17496/kmer.23.014.

[20] Yim MK. Reforms of the Korean Medical Licensing examination regarding item development and performance evaluation. J Educ Eval Health Prof. 2015;12:6. 10.3352/jeehp.2015.12.6. 25797057 10.3352/jeehp.2015.12.6 PMC4397843

[21] Van Manen M. Phenomenology of practice: meaning-giving methods in Phenomenological Research and writing. Walnut Creek (CA): Left Coast; 2014.

[22] Larkin M, Flowers P, Smith JA. Interpretative phenomenological analysis: theory, method and research. London, UK: Sage; 2009.

[23] Husserl E. Ideas: General introduction to pure phenomenology. London, UK: Routledge; 2012.

[24] Jeong H, Othman J. Using interpretative phenomenological analysis from a realist perspective. Qualitative Rep. 2016;21(3):558–70.

[25] The Korean Society of Medical Education. Awards. https://www.ksmed.or.kr/html/?pmode=awards. Accessed 8 April 2024.

[26] Korea Association of Medical College. Bulletin Board (Notice). https://www.kamc.kr/main/index.php?m_cd=14&b_id=2017052218150374. Accessed 8 April 2024.

[27] Denzin NK. The research act: a theoretical introduction to sociological methods. 2nd ed. New York (NY): McGraw Hill; 1978.

[28] Van Nes F, Abma T, Jonsson H, Deeg D. Language differences in qualitative research: is meaning lost in translation. Eur J Ageing. 2010;7(4):313–6. 10.1007/s10433-010-0168-y. 21212820 10.1007/s10433-010-0168-y PMC2995873

[29] Behr D. Assessing the use of back translation: The shortcomings of back translation as a quality testing method. International Journal of Social Research Methodology. 2017;20(6):573–584. 10.1080/13645579. 2016.1252188.

[30] Santos HP Jr, Black AM, Sandelowski M. Timing of translation in cross-language qualitative research. Qual Health Res. 2015;25(1):134–44. 10.1177/1049732314549603. 25189538 10.1177/1049732314549603

[31] The career is dead -. Long live the Career: a Relational Approach to Careers, Jossey-Bass, San Francisco, CA.

[32] Hall DT. The protean career: a quarter-century journey. J Vocat Behav. 2004;65(1):1–13.

[33] Pulakos ED, Arad S, Donovan MA, Plamondon KE. Adaptability in the workplace: development of a taxonomy of adaptive performance. J Appl Psychol. 2000;85(4):612–24. 10948805 10.1037/0021-9010.85.4.612

[34] Bandura A. Social cognitive theory: an agentic perspective. Annu Rev Psychol. 2001;52:1–26. 11148297 10.1146/annurev.psych.52.1.1

[35] Seibert SE, Kraimer ML. The five-factor model of personality and Career Success. J Vocat Behav. 2001;58(1):1–21.

[36] Dries N, Pepermans R, Carlier O. Career success: constructing a multidimensional model. J Vocat Behav. 2008;73(2):254–67.

[37] Heslin PA. Conceptualizing and evaluating career success. J Organizational Behav. 2005;26(2):113–36.

[38] Srinivasan M, Li S-TT, Meyers FJ, Pratt DD, Collins JB, Braddock C, et al. Teaching as a competency: competencies for medical educators. Acad Med. 2011;86(10). 10.1097/ACM.0b013e31822c5b9a. 10.1097/ACM.0b013e31822c5b9a 21869655

[39] De Vos A, Soens N. Protean attitude and career success: the mediating role of self-management. J Vocat Behav. 2008;73(3):449–56.

[40] Waters L, Briscoe JP, Hall DT, Wang L. Protean career attitudes during unemployment and reemployment: a longitudinal perspective. J Vocat Behav. 2014;84(3):405–19.

[41] Supeli A, Creed PA. The longitudinal relationship between Protean Career Orientation and Job satisfaction, Organizational Commitment, and intention-to-quit. J Career Dev. 2016;43(1):66–80.

[42] Karimi E, Sohrabi Z, Aalaa M. Change Management in Medical contexts, especially in Medical Education: a systematized review. J Adv Med Educ Prof. 2022;10(4):219. 10.30476/JAMP.2022.96519.1704. 36310665 10.30476/JAMP.2022.96519.1704 PMC9589067

[43] Maaz A, Hitzblech T, Arends P, et al. Moving a mountain: practical insights into mastering a major curriculum reform at a large European medical university. Med Teach. 2018;40(5):453–60. 10.1080/0142159X.2018.1440077. 29504437 10.1080/0142159X.2018.1440077

[44] Nicholson N, Wendy de Waal Andrews. Playing to Win: Biological imperatives, Self-Regulation, and Trade-offs in the game of Career Success. J Organizational Behav. 2005;26(2):137–54. 10.1002/job.295.

[45] Skjevik EP, Schei E, Boudreau JD, et al. What makes mentors thrive? An exploratory study of their satisfaction in undergraduate medical education. BMC Med Educ. 2024;24(1):372. 10.1186/s12909-024-05344-y. 38575953 10.1186/s12909-024-05344-y PMC10996132

[46] Stenfors-Hayes T, Kalén S, Hult H, Dahlgren LO, Hindbeck H, Ponzer S. Being a mentor for undergraduate medical students enhances personal and professional development. Med Teach. 2010;32(2):148–53. 10.3109/01421590903196995. 20163231 10.3109/01421590903196995

[47] Gerrity MS, Pathman DE, Linzer M, Steiner BD, Winterbottom LM, Sharp MC, Skochelak SE. Career satisfaction and clinician-educators. The rewards and challenges of teaching. The Society of General Internal Medicine Career Satisfaction Study Group. J Gen Intern 0Med. 1997;12(Suppl 2):S90–7. 10.1046/j.1525-1497.12.s2.13.x. 10.1046/j.1525-1497.12.s2.13.x PMC1497234 9127250

[48] Harden RM. International medical education and future directions: a global perspective. Acad Med. 2006;81(12 Suppl):S22–9. 10.1097/01.ACM.0000243411.19573.58. 17086041 10.1097/01.ACM.0000243411.19573.58

[49] Meyer JP, Stanley DJ, Herscovitch L, Topolnytsky L. Affective, continuance, and normative commitment to the organization: a meta-analysis of antecedents, correlates, and consequences. J Vocat Behav. 2003;61(1):20–52. 10.1006/jvbe.2001.1842.

[50] Meyer JP, Becker TE, Vandenberghe C. Employee commitment and motivation: a conceptual analysis and integrative model. J Appl Psychol. 2004;89(6):991–1007. 10.1037/0021-9010.89.6.991. 15584837 10.1037/0021-9010.89.6.991

[51] Riketta M. Organizational identification: a meta-analysis. J Vocat Behav. 2005;66(2):358–84.

[52] Schmidt KH. Organizational commitment: a further moderator in the relationship between work stress and strain? Int J Stress Manage. 2007;14(1):26–40. 10.1037/1072-5245.14.1.26.

[53] Heslin PA. Conceptualizing and evaluating career success. J Organizational Behav. 2005;26(2):113–36. 10.1002/job.270.

[54] Bartlett KR. The relationship between training and organizational commitment: a study in the health care field. Hum Res Dev Q. 2001;12(4):4:335–52. 10.1002/hrdq.1001.

[55] Lee DWC, Tan CKN, Tan K, Yee XJ, Jion Y, Roebertsen H, Dong C. How community and organizational culture interact and affect senior clinical educator identity. Med Teach. 2024;46(4):564–72. 10.1080/0142159X.2023.2262103. 37813120 10.1080/0142159X.2023.2262103

