# Supplementary material for: Navigating protean career paths in medical education: insights from outstanding medical educators in South Korea
Source: BMC Med Educ. 2024 Oct 19;24:1174. doi: 10.1186/s12909-024-06183-7 (PMC11490113; doi:10.1186/s12909-024-06183-7)
Supplement: Supplementary file 1 — Supplementary Material 1 [file 12909_2024_6183_MOESM1_ESM.docx]

**Appendix1. Questions used for the semi-structured interviews.**

| **No.** | **Questions** |
| --- | --- |
| 1 | What was the trigger or event that got you interested or involved in medical education, and what did that trigger, or event mean to you? |
| 2 | Can you describe your career path in medical education?  In your experience to date, what has been the most meaningful activity in medical education? What changes have you sought through your activities in medical education and why were they meaningful to you? |
| 3 | What competencies did you find necessary to be active in medical education, how did you go about developing those competencies, and do you think those methods were effective? |
| 4 | What organizational support or culture do you think is necessary for your work in medical education? |
| 5 | How important do you think internal and external networks are to your work in medical education? Tell us about your experience building or changing networks. |
| 6 | What activities or roles do you think medical educators will be expected to perform in the future? What do you think are the key elements of career development to be successful in these activities or roles? |
| 7 | What are your overall recommendations for career development for the next generation of medical educators? |
